# The Sodium-Glucose Cotransporter 2 Inhibitor Dapagliflozin Prevents Renal and Liver Disease in Western Diet Induced Obesity Mice

**DOI:** 10.3390/ijms19010137

**Published:** 2018-01-03

**Authors:** Dong Wang, Yuhuan Luo, Xiaoxin Wang, David J. Orlicky, Komuraiah Myakala, Pengyuan Yang, Moshe Levi

**Affiliations:** 1 Institutes of Biomedical Sciences and Department of Chemistry, Fudan University, Shanghai 200032, China; dong.2.wang@ucdenver.edu (D.W.); pyyang@fudan.edu.cn (P.Y.); 2 Department of Renal & Hypertension, Department of Pediatrics and Department of Pathology, University of Colorado Denver, Anschutz Medical Campus, 12800 19th Ave., Aurora, CO 80045, USA; yuhuan.luo@ucdenver.edu (Y.L.); xiaoxin.wang@gergeotown.edu (X.W.); david.orlicky@ucdenver.edu (D.J.O.); komuraiah.myakala@georgetown.edu (K.M.); 3 Department of Biochemistry and Molecular & Cellular Biology, Georgetown University, Washington, DC 20072, USA; 4 Department of Systems Biology for Medicine, Basic Medical College, Fudan University, Shanghai 200032, China

**Keywords:** obesity, SGLT2, dapagliflozin, fibrosis, lipid, inflammation

## Abstract

Obesity and obesity related kidney and liver disease have become more prevalent over the past few decades, especially in the western world. Sodium-glucose cotransporter 2 (SGLT2) inhibitors are a new class of antidiabetic agents with promising effects on cardiovascular and renal function. Given SGLT2 inhibitors exert both anti-diabetic and anti-obesity effects by promoting urinary excretion of glucose and subsequent caloric loss, we investigated the effect of the highly selective renal SGLT2 inhibitor dapagliflozin in mice with Western diet (WD) induced obesity. Low fat (LF) diet or WD-fed male C57BL/6J mice were treated with dapagliflozin for 26 weeks. Dapagliflozin attenuated the WD-mediated increases in body weight, plasma glucose and plasma triglycerides. Treatment with dapagliflozin prevented podocyte injury, glomerular pathology and renal fibrosis determined by second harmonic generation (SHG), nephrin, synaptopodin, collagen IV, and fibronectin immunofluorescence microscopy. Oil Red O staining showed dapagliflozin also decreased renal lipid accumulation associated with decreased SREBP-1c mRNA abundance. Moreover, renal inflammation and oxidative stress were lower in the dapagliflozin-treated WD-fed mice than in the untreated WD-fed mice. In addition, dapagliflozin decreased serum alanine aminotransferase (ALT) and aspartate aminotransferase (AST), hepatic lipid accumulation as determined by H&E and Oil Red O staining, and Coherent Anti-Stokes Raman Scattering (CARS) microscopy, and hepatic fibrosis as determined by picrosirius red (PSR) staining and TPE-SHG microscopy in WD-fed mice. Thus, our study demonstrated that the co-administration of the SGLT2 inhibitor dapagliflozin attenuates renal and liver disease during WD feeding of mice.

## 1. Introduction

Obesity has become much more prevalent around the world over the past few decades. There is strong association between the obesity and diabetes mellitus [1,2]. Being overweight or obese increases the chances of having type 2 diabetes mellitus (type 2 DM). Excess weight is also an important risk factor for essential hypertension and end-stage renal disease (ESRD). One of the main mechanisms through which obesity increases the risk of diabetes is abdominal fat driven metabolic complications that lead to glucose intolerance, dyslipidemia, hyperinsulinemia, and insulin resistance [3], which are important components in the development of type 2 DM. Obesity also results in increased blood pressure, renal vasodilation and glomerular hyperfiltration [4,5] which cause a hemodynamic burden on the kidneys and can lead to kidney injury. Overnutrition also carries an increased risk of liver disease. Both cirrhosis and hepatocellular carcinoma can be sequela of obesity. In patients with nonalcoholic fatty liver disease (NAFLD) or nonalcoholic steatohepatitis (NASH), obesity is highly associated with advanced fibrosis [6].

Type 2 DM is the most common form of diabetes and accounts for 90–95% of all diabetes cases. It continues to be the leading cause of renal disease in the U.S. and around the world [7,8]. In diabetic kidney disease (diabetic nephropathy) glomerular cells become damaged resulting in increased albumin loss. Despite all the beneficial interventions implemented in patients with diabetes, including tight glucose control and tight blood pressure control with regimens that inhibit the renin angiotensin aldosterone system, renal disease still progresses in most diabetic patients. Diabetes is associated with a series of liver diseases including nonalcoholic fatty liver disease (NAFLD), nonalcoholic steatohepatitis (NASH), cirrhosis, fulminant hepatic failure and hepatocellular carcinoma [9,10].

It is well-known that the proximal tubular glucose absorption is increased in Type 2 DM humans and in animal models of diabetes mellitus [11]. The sodium-glucose linked cotransporters, SGLT, are a family of glucose transporters that contribute to renal glucose reabsorption [12]. SGLT1 is mainly expressed in the gastrointestinal tract and to some extent in the kidney proximal straight tubule and is a major transporter of dietary glucose and galactose, while SGLT2 is highly expressed in the kidney proximal convoluted tubule [13,14,15]. SGLT1 and SGLT2 knock out mouse studies determined that 97% of proximal tubular glucose transport is mediated via SGLT2 and only 3% by SGLT1 [16,17]. SGLT-2 inhibitors are a new class of antidiabetic drugs which inhibit renal glucose reabsorption [18,19]. Inhibition of SGLT2 results in a decrease in blood glucose due to increased renal glucose excretion. SGLT2 knock-out studies in mice with streptozotocin-induced hyperglycemia showed that absence of SGLT2 attenuated glomerular hyperfiltration and hyperglycemia [20]. In type 2 diabetic mice, treatment with SGLT2 inhibitors decreased plasma glucose, albuminuria, mesangial expansion and glomerular hypertrophy, and inhibited inflammation and oxidative stress [21,22,23,24].

Dapagliflozin is a highly selective inhibitor of renal SGLT2, which is used in the management of patients with type 2 diabetes [25,26,27]. Numerous well-designed clinical trials and animal model studies have demonstrated that dapagliflozin significantly reduces fasting and postprandial blood glucose and glycosylated hemoglobin (HbA1c) [28]. Besides its antidiabetic effect, dapagliflozin has also been found to prevent the development of nephropathy, probably by decreasing body weight, uric acid, blood pressure, lipid metabolism, inflammation and improving insulin resistance. A study in high-fat fed diabetic mice indicated that dapagliflozin might be of therapeutic potential for diabetic atherosclerosis via anti-inflammatory effects [29]. In addition, dapagliflozin has been shown to slow the progression of diabetes-associated glomerulosclerosis and liver fibrosis by improving hyperglycemia-induced tissue inflammation and oxidative stress [30].

Dramatic weight loss has been observed in clinical trials and diabetic animal models, which is the result of reduced adipose tissue stores caused by energy excretion as glucose. However, the effect of dapagliflozin in an obesity model has not been well studied. It is also important to further characterize the potential mechanisms of dapagliflozin in liver and kidney disease associated with obesity.

In our previous studies, we have determined that treatment of *db*/*db* mice, a model of type 2 DM, with the SGLT-2 inhibitor JNJ 39933673 resulted in control of blood glucose and triglyceride levels. In addition, there was a marked reduction in albuminuria, glomerular mesangial expansion and extracellular matrix protein accumulation, and podocyte loss [31]. These beneficial effects were associated with marked decreases in renal lipid accumulation, inflammation, oxidative stress, and profibrotic growth factors.

The aims of the present study were to explore the effects of dapagliflozin on the progression of renal and liver disease in mice fed a Western diet supplemented or not with dapagliflozin. We found that dapagliflozin supplementation of the western diet attenuated the progression of nephropathy and liver disease by inhibiting lipid accumulation, inflammation, and fibrosis.

## 2. Results

### 2.1. Dapagliflozin Treatment of WD-Fed C57BL/6J Mice Reduced Body Weight Gain and Improved Plasma Glucose, Insulin Resistance and Plasma Lipids

**Figure 1:**
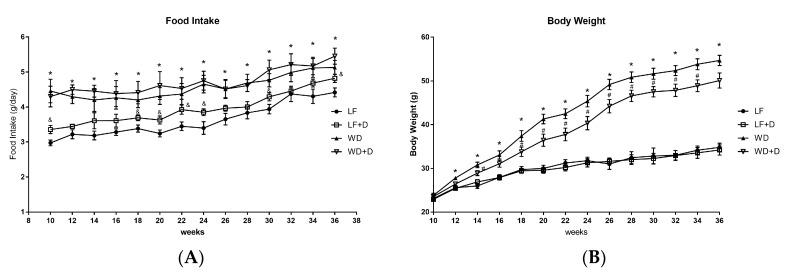
Effect of western diet and dapagliflozin on body weight and food intake. (**A**) Body weight was higher in the WD-fed group than in low fat group during the study. Body weight in the WD-fed with dapagliflozin group was lower than in the WD-fed group from 10 to 36 weeks of age. (**B**) Food intake in WD + dapagliflozin group was slightly higher than in the WD group. Results are expressed as means ± SEM (*n* = 6 mice per experimental and treatment group). Statistical analysis was performed with one-way ANOVA. * vs. low fat group (*p* < 0.05), # vs. Western diet group (*p* < 0.05), & LF + dapagliflozin group vs. LF group (*p* < 0.05).

In order to determine the effects with dapagliflozin on WD-induced obesity and insulin resistance, C57BL/6J mouse were fed with a low-fat diet (LF), a Western diet (WD), LF with dapagliflozin, and WD with dapagliflozin. The studies were started when the mice were 10 weeks old and the mice were treated for 26 weeks ending when they were 36 weeks old. Body weight of above experimental groups were measured throughout the treatment period. The growth curve showed that there was significant weight gain in WD-fed mice compared with LF-fed mice. Dapagliflozin treatment provided a degree of protection from weight gain, decreasing it significantly in the WD plus dapagliflozin group but not in the LF plus dapagliflozin group (Figure 1A). Body weight, liver weight, fasting plasma triglycerides, cholesterol and insulin levels all were significantly increased in WD-fed mice, while treatment with dapagliflozin markedly reversed these increases (Table 1). The WD-fed mice consumed more food than LF-fed mice, but there was no change in food intake between dapagliflozin-treated WD-fed mice and WD-fed mice throughout the treatment period (Figure 1B). As expected, SGLT2 inhibition also decreased the plasma glucose but it did not normalize it in these studies. No significant change was found in systolic blood pressure among these groups.

### 2.2. Dapagliflozin Prevented Glomerular Pathology and Renal Fibrosis in WD-Fed Mice

**Figure 2:**
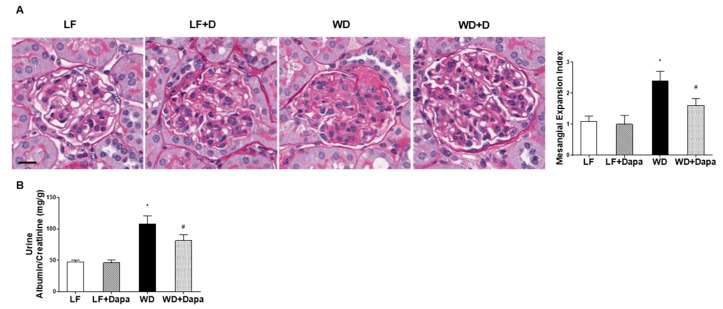
Dapagliflozin prevented glomerular pathology and increased urinary albumin excretion in WD-fed mice. (**A**) Representative periodic acid-Schiff (PAS) staining of kidney sections. Mesangial expansion index was defined as the percentage of mesangial area in glomerular tuft area. The mesangial area was determined by assessment of PAS-positive and nucleus-free areas in the mesangium. Scale bar = 30 µm; (**B**) Urine albumin vs. creatinine ratio. Results are expressed as means ± SEM (*n* = 6 mice per group). Statistical analysis was performed with one-way ANOVA. * vs. low fat group (*p* < 0.05), # vs. Western diet group (*p* < 0.05).

To evaluate the effects of WD-induced obesity and insulin resistance on renal histopathology, we performed periodic acid-Schiff (PAS) staining on kidney sections. Compared with C57BL/6J mice fed low fat diet, kidney sections of C57BL/6J mice fed WD showed increased mesangial expansion (Figure 2A). This outcome was ameliorated in the group of mice fed WD plus dapagliflozin. The glomerular changes in C57Bl/6J mice fed WD were also associated with a significant increase in urinary albumin excretion, which is an indicator of glomerular injury with loss of glomerular permselectivity. The increased albuminuria was prevented by treatment with dapagliflozin (Figure 2B).

**Figure 3:**
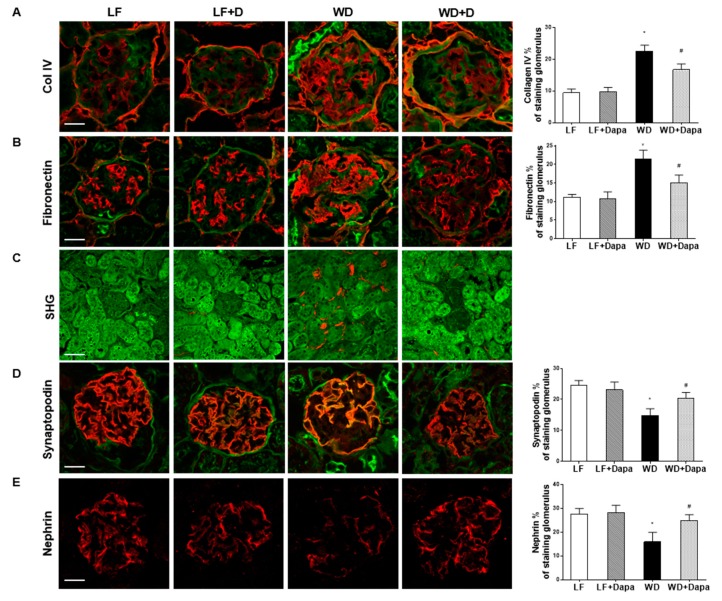
Dapagliflozin prevented glomerular pathology and renal fibrosis in WD-fed mice. Shown are representative images of Immunofluorescence staining of kidney sections for (**A**) collagen IV; (**B**) Fibronectin; (**C**) Representative merged two-photon excitation (green)-SHG (red) images of kidney sections; (**D**) synaptopodin; and (**E**) nephrin. Scale bar = 20 µm in A, B, D and E, 100 µm in C. Results are expressed as means ± SEM (*n* = 6 mice per group). Statistical analysis was performed with one-way ANOVA. * vs. low fat group (*p* < 0.05), # vs. Western diet group (*p* < 0.05).

We found dapagliflozin could prevent the increases in extracellular matrix accumulation in WD-fed mice as determined by type IV collagen and fibronectin immunofluorescence microscopy (Figure 3A,B). Picrosirius red staining showed that Western diet treatment slightly increased the collagen aground many of the tubules in the outer stripe of the outer medulla compared to low fat diet fed group, but it did not reveal significant changes among all groups (Figure S1). Highly ordered fibrillary collagens (types I and III) produce SHG signals, which can be visualized in tissue without the need for exogenous labeling. We then used label-free and stain-free imaging with Two Photon Excitation (TPE)-Second Harmonic Generation (SHG) Microscopy to visualize the fibrillary structure in paraffin-embedded and unstained kidney sections. SHG revealed accumulation of extracellular matrix proteins in the tubular interstitium in WD-fed mice which was attenuated by treatment with dapagliflozin (Figure 3C). 

Staining for the presence of nephrin, a podocyte marker, showed a significant decrease in WD-fed C57BL/6J mice compared with LF-fed mice. Similarly, the presence of synaptopodin another podocyte marker was also decreased in the WD group. SGLT2 inhibition by dapagliflozin prevented podocyte injury and loss of podocytes as determined by IF staining for synaptopodin and nephrin (Figure 3D,E).

### 2.3. Dapagliflozin Decreased Lipid Accumulation in Kidney

**Figure 4:**
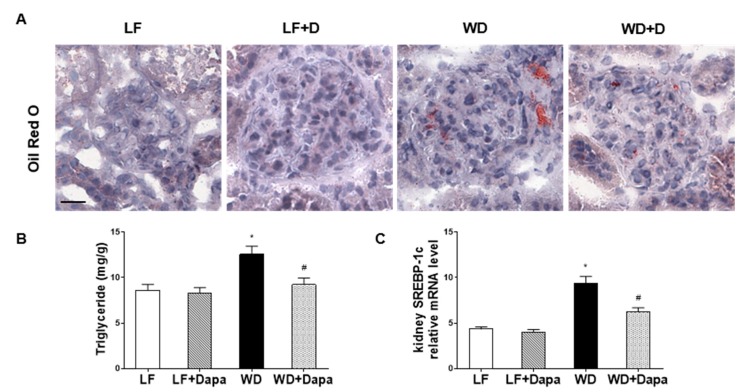
Dapagliflozin decreased renal lipid accumulation in WD-fed mice. (**A**) Oil red O staining of kidney sections. Scale bar = 20 µm; (**B**) Kidney triglyceride content analysis; (**C**) Quantitative real-time PCR (qRT-PCR) analysis of fatty acid synthesis master gene (SREBP-1c). Results are expressed as means ± SEM (*n* = 6 mice per group). Statistical analysis was performed with one-way ANOVA. * vs. low fat group (*p* < 0.05), # vs. Western diet group (*p* < 0.05).

In a previous study we showed that treatment with another SGLT2 inhibitor was able to decrease renal lipid accumulation in *db*/*db* mice [31]. Altered regulation of renal lipid metabolism has been shown to play an important role in the pathogenesis of diet-induced, obesity-related kidney disease [31,32,33]. To study whether dapagliflozin could decrease renal lipid accumulation, Oil Red O staining was performed to reveal the accumulation of neutral lipids (triglycerides and cholesterol ester) in the glomerular and tubulointerstitial cells. There was a significant increase in renal triglyceride and cholesterol content in WD-fed mice (Figure 4B), which correlated directly with increased Oil Red O staining (Figure 4A). Treatment with dapagliflozin significantly decreased lipid accumulation in WD-fed mice (Figure 4A). These changes were associated with a significant decrease in kidney triglyceride level (Figure 4B), while there was only a slight reduction of total kidney cholesterol level in dapagliflozin treatment mice. Furthermore, dapagliflozin reduced the mRNA expression of the fatty acid and triglyceride synthesis master transcription factor SREBP-1c in WD-fed mice (Figure 4C). These results indicate that treatment of dapagliflozin decreases the renal triglyceride level by inhibiting fatty acid and triglyceride synthesis pathways.

### 2.4. Dapagliflozin Decreased Renal Inflammation and Oxidative Stress in WD-Fed Mice

**Figure 5:**
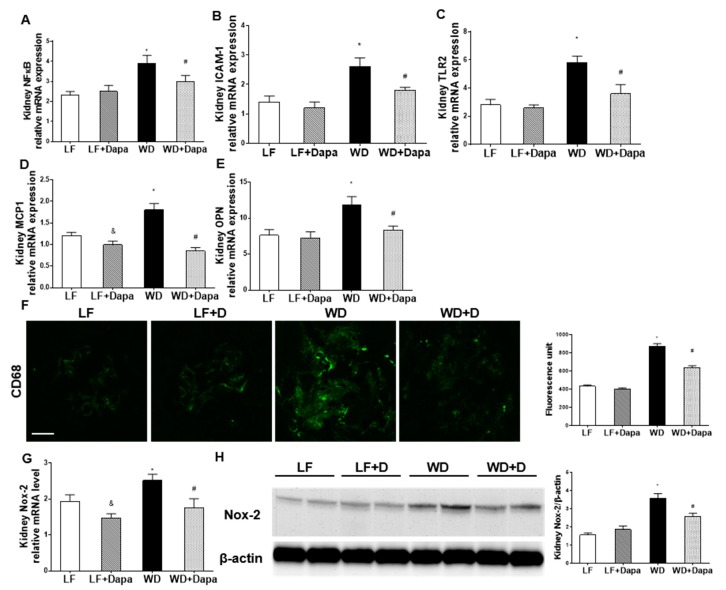
Dapagliflozin decreased renal inflammation and oxidative stress in WD-fed mice. (**A**–**E**) Quantitative real-time PCR (qRT-PCR) analysis of nuclear factor kappa-light-chain-enhancer of activated B cells (NFκB), intercellular adhesion molecule-1 (ICAM-1), toll-like receptor 2 (TLR2), monocyte chemotactic protein-1 (MCP-1) and osteopontin (OPN); (**F**) Immunofluorescence staining of kidney sections for CD68 (green) and the quantification of the green florescence. Scale bar = 20 µm; (**G**) Quantitative real-time PCR (qRT-PCR) analysis of Nox-2; (**H**) Western blot of Nox-2 in kidney and quantification of the observed levels. Equal protein loading and transfer was verified by using an anti-β-actin antibody. The detection was performed by an ECL western blotting analysis system. Results are expressed as means ± SEM (*n* = 6 mice per group, *n* = 4 for western blotting). Statistical analysis was performed with one-way ANOVA. * vs. low fat group (*p* < 0.05), # vs. Western diet group (*p* < 0.05), & LF + dapagliflozin group vs. LF group (*p* < 0.05).

Renal inflammation and oxidative stress are characteristic findings of both obesity and metabolic syndrome. There were significant increases in the kidney mRNA abundance of the inflammatory markers, nuclear factor kappa-light-chain-enhancer of activated B cells (NFκB), intercellular adhesion molecule-1 (ICAM-1), toll-like receptor 2 (TLR2), monocyte chemotactic protein-1 (MCP-1) and osteopontin (OPN), in WD-fed mice compared with LF-fed mice (Figure 5A–E). The mRNA abundance of those genes was significantly lower in dapagliflozin treated mice compared to the WD-fed mice. In addition, dapagliflozin decreased the IF expression of the macrophage marker CD68 in WD-fed obesity mice (Figure 5F). Dapagliflozin also prevented oxidative stress in the kidney by decreasing NADPH oxidase (Nox-2) mRNA and immunoblot detectable Nox-2 protein (Figure 5G,H).

### 2.5. Dapagliflozin Reduced Hepatic Injury and Lipid Accumulation in Liver

**Figure 6:**
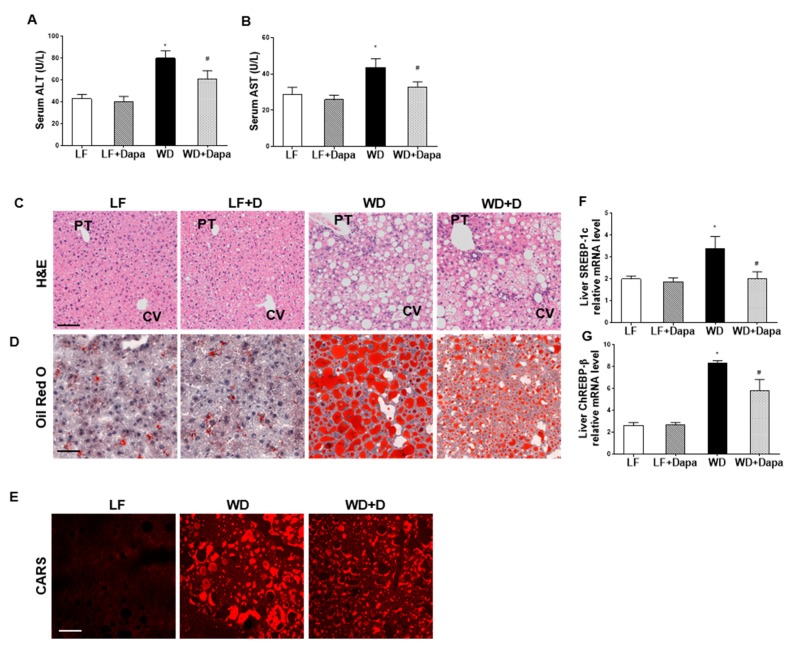
Dapagliflozin reduced hepatic injury and lipid accumulation in liver of WD-fed mice. (**A**,**B**) Serum ALT and AST activity; (**C**) H&E staining, CV, central vein and PT, portal triad; (**D**) Oil red O staining of liver sections; (**E**) lipid droplets were visualized by Coherent Anti-Stokes Raman Scattering (CARS) Microscopy of liver sections; (**F**,**G**) Quantitative real-time PCR (qRT-PCR) analysis of fatty acid synthesis master gene (SREBP-1c) and carbohydrate-responsive element-binding protein-β (ChREBP-β). Scale bars = 100 µm. Results are expressed as means ± SEM (*n* = 6 mice per group). Statistical analysis was performed with one-way ANOVA. * vs. low fat group (*p* < 0.05), # vs. Western diet group (*p* < 0.05).

WD-fed mice had significantly increased plasma alanine aminotransaminase (ALT) and aspartate aminotransferase (AST) compared with LF-fed mice. Treatment of WD-fed mice with dapagliflozin ameliorated the elevated ALT and AST (Figure 6A,B).

H&E staining of liver sections showed intracellular lipid accumulation, ballooning, and inflammation with increases in hepatic lipid content and steatosis in WD-fed mice. Dapagliflozin treatment reduced these changes (Figure 6C). Oil Red O staining indicated that there was a marked increase in lipid droplets accumulated in the liver of WD-fed mice, while many fewer lipid droplets were found in LF-fed groups. The number and size of the hepatic lipid droplets were significantly decreased by dapagliflozin treatment in the WD-fed group (Figure 6D). Label-free imaging with CARS Microscopy also revealed increased lipid accumulation in the livers of mice fed WD when compared to LF fed mice. Similarly, dapagliflozin treatment markedly decreased the size and intensity of the CARS positive lipid droplets (Figure 6E).

To explore the mechanism by which SGLT2 inhibition regulates liver lipid metabolism, we examined two of the important transcription factors that regulate lipogenesis. We found that the expression of the master fatty acid synthesis transcription factors sterol regulatory element binding protein-1c (SREBP-1c) and carbohydrate-responsive element-binding protein-β (ChREBP-β) were reduced following treatment with dapagliflozin. Therefore, treatment with dapagliflozin inhibited fatty acid synthesis and triglyceride pathways resulting in decreased liver triglyceride content (Figure 6F,G). 

Our results showed that hepatic lipid accumulation was significantly increased in C57BL/6J mice fed a Western diet compared to LF-fed mice, and dapagliflozin treatment significantly decreased the lipid accumulation in mice fed the Western diet.

### 2.6. Dapagliflozin Attenuates Obesity-Induced Inflammation and Oxidative Stress in Liver

**Figure 7:**
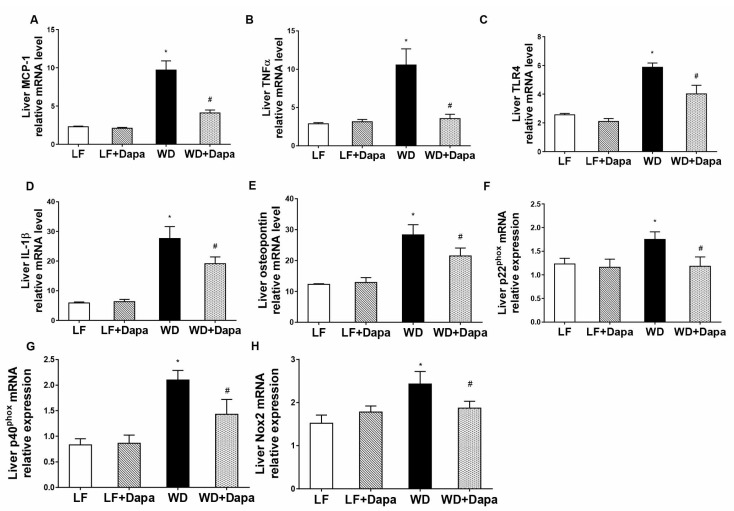
Dapagliflozin decreased inflammation in the liver of WD-fed mice. (**A**–**E**) Quantitative real-time PCR (qRT-PCR) analysis of monocyte chemoattractant protein-1 (MCP1), tumor necrosis factor alpha (TNFα), Toll-like receptor 4 (TLR4), interleukin-1 beta (IL-1β) and Osteopontin (OPN) in liver. Results are expressed as means ± SEM (*n* = 6 mice per group). Statistical analysis was performed with one-way ANOVA. * vs. low fat group (*p* < 0.05), # vs. Western diet group (*p* < 0.05).

To determine whether the WD induced liver inflammation and whether dapagliflozin could attenuate that inflammation, interleukin-1β (IL-1β), tumor necrosis factor alpha (TNFα), Toll-like receptor 4 (TLR4), Osteopontin (OPN) and monocyte chemoattractant protein-1 (MCP1) mRNA abundance were measured (Figure 7A–E). The mRNA abundance of all of those genes was significantly increased in WD-fed mice compared with LF-fed mice, and importantly, dapagliflozin markedly suppressed the WD induced increases. In addition, the increased mRNA expression of the subunits of NADPH oxidase p22phox, p40phox and Nox2 in WD-fed mice was also decreased by dapagliflozin (Figure 7F–H). These results indicate that WD-induced obesity increased inflammation in liver tissue, which was attenuated by dapagliflozin treatment.

### 2.7. Dapagliflozin Decreases Fibrosis in Liver

**Figure 8:**
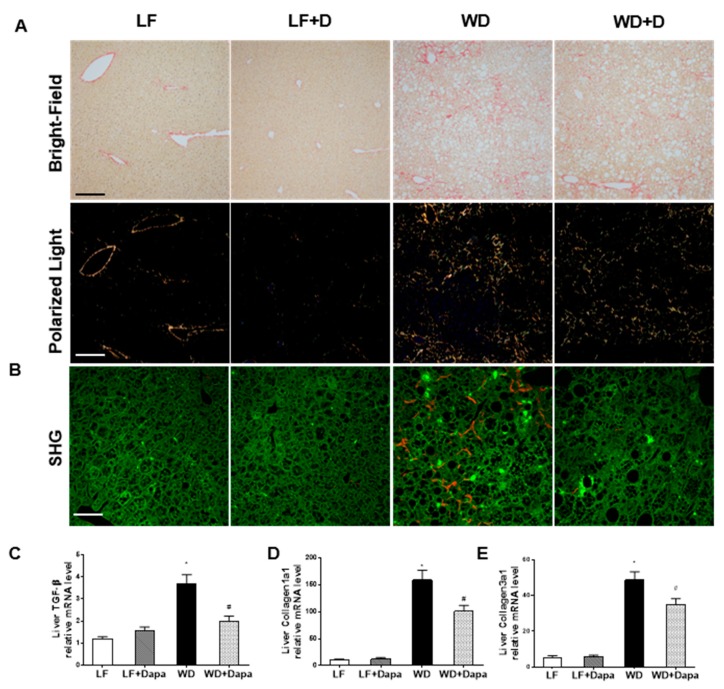
Dapagliflozin decreased liver fibrosis in WD-fed obesity mice. (**A**) Picro Sirius Red staining for fibrillar collagens in liver sections, when visualized using either brightfield or polarized light microscopy of the same field; (**B**) Representative merged two-photon excitation (green)-SHG (red) images of liver sections; (**C**–**E**) Quantitative real-time PCR (qRT-PCR) analysis of profibrotic factor TGF-β, collagen1a1 and collagen3a1 gene expression in the liver. Scale bars = 200 µm in (**A**), 100 µm in (**B**). Results are expressed as means ± SEM (*n* = 6 mice per group). Statistical analysis was performed with one-way ANOVA. * vs. low fat group (*p* < 0.05), # vs. Western diet group (*p* < 0.05).

Liver fibrosis was quantified by Picro Sirius red staining followed by polarized-light imaging as well as TPE-SHG microscopy. LF-fed mouse displayed no appreciable alterations in histology, and had an almost negligible amount of fibrous tissue, however, in the WD-fed group, there was an increased accumulation of fibrillar collagen. (Figure 8A,B). The increase in fibrosis was associated with significant increases in the mRNA abundance of profibrotic TGF-β, Col1a1, and Col3a1 (Figure 8C–E). Dapagliflozin treatment ameliorated those outcomes to a large degree in WD-fed mice.

## 3. Discussion

Sodium-glucose cotransporter 2 (SGLT2) inhibitors are a new class of antidiabetic agents with promising effects on prevention of diabetic renal disease [34]. We have previously reported that SGLT2 mRNA and protein expression are increased in human biopsies of diabetic nephropathy even with advanced kidney disease indicating that SGLT2 can be an effective target in treatment of diabetic nephropathy. We also showed that SGLT2 inhibition decreases renal lipid accumulation and inflammation and prevents the development of nephropathy in *db*/*db* mice regardless whether they have insulin-deficient diabetes or insulin-resistant diabetes [31].

Clinical trials and animal studies have reported that the beneficial effects of dapagliflozin are associated with increased urinary glucose and sodium excretion, leading to blood glucose reductions and weight loss in type 2 diabetes or obesity [31,35,36]. In the present study, we demonstrated the protective effects of the SGLT2 inhibitor, dapagliflozin on nephropathy in mice with Western diet induced obesity. Administration of dapagliflozin prevented glomerular changes and renal fibrosis determined by PAS staining, SHG microscopy, PSR staining and Collagen IV, nephrin, synaptopodin, fibronectin IF microscopy. Oil Red O staining showed dapagliflozin also decreased renal lipid accumulation which paralleled a decreased expression of SREBP-1c transcripts. Moreover, renal inflammation and oxidative stress were lower in the dapagliflozin-treated WD-fed mice than in the untreated obese mice. Body weight reduction was found in dapagliflozin treated mice, but we did not find a significant alteration of food intake following treatment with dapagliflozin. The observed weight loss appeared to be a function of reduced adipose mass which resulted from the SGLT2 inhibitor-decreased glucose level thus enhancing fat utilization. All of the beneficial effects appeared to be mediated by normalization of serum glucose. In summary, these findings revealed that dapagliflozin exhibits the effects on ameliorating the progression of obesity related nephropathy.

Obesity can cause fatty deposits to build up in the liver (hepatosteatosis). Nonalcoholic fatty liver disease (NAFLD), nonalcoholic steatohepatitis (NASH), liver fibrosis, and cirrhosis are highly associated with obesity [37,38]. A previous study reported metabolic effects of SGLT2 inhibition on hepatic steatosis in a type 2 diabetic mouse model. In that study, treatment with dapagliflozin for 4 weeks markedly decreased the elevated liver enzyme activity, fat content as well as liver inflammation, oxidative stress and fibrosis [30]. Ipragliflozin improved the pathogenesis of NASH by reducing insulin resistance and lipotoxicity [39]. Tofogliflozin suppressed high fat diet induced weight gain and hepatic steatosis [40], while remogliflozin reduced hepatic steatosis without affecting weight gain in the HFD fed animal [41]. However, the effects of SGLT2 inhibition by dapagliflozin in WD-induced obesity-related liver disease have not been reported yet. In the present study, we found dapagliflozin reduced hepatic injury and lipid accumulation histologically as well while utilizing, Oil Red O staining and CARS microscopy in WD-fed mice. Alanine aminotransferase (ALT), which indicates liver inflammation or injury, is a candidate marker for NAFLD and NASH. In recent years, it has been reported that ALT is a useful indicator of the progression of liver fibrosis in NAFLD patients [42]. Dapagliflozin significantly reduced the elevated level of ALT in obese mice, while a markedly reduction of AST level was also observed. Reduction of the markers of inflammation including mRNA levels of TNFα, TLR4, OPN, MCP1 and IL-1β, as well as fibrosis including mRNA levels of TGF-β, Col1a1, and Col3a1 in liver were also observed. Furthermore, Picro Sirius red staining and SHG microscopy also showed a significant decrease in liver fibrosis with dapagliflozin treated WD-fed mice.

It appears that when glucose is lost through urinary excretion processes, the mouse’s whole-body metabolism undergoes an adaptive change involving glucose fluxes, hormonal responses, and fuel selection, all of which are key to affect hepatic function. Lipotoxicity is excess accumulation of lipids in non-adipose tissues and is caused by an increase in the flux of free fatty acid within hepatocytes. Previous studies demonstrated that an excess accumulation of lipids leads to cellular injury and death and that this is one of the key factors of inflammation and fibrosis [43]. Liver fibrosis is a marker of liver disease progression. Our findings showed that dapagliflozin helps prevent liver lipid inflammation and fibrosis as a result of decreased lipid accumulation.

In conclusion, we identified a potent effect of the SGLT2 inhibitor dapagliflozin on reducing obesity in Western diet fed C57BL/6J mice. Dapagliflozin prevents glomerular changes, fibrosis, inflammation, oxidative stress and lipid accumulation in the kidneys of WD-fed mice, and attenuates obesity-induced inflammation, fibrosis, and lipid accumulation in the liver of WD-fed mice. Overall, the current study highlights the potential clinical utility of SGLT2 inhibition in the prevention of obesity related kidney and liver disease. 

## 4. Materials and Methods

### 4.1. Animal Models

Ten-week-old male C57BL/6J mice were obtained from the Jackson Laboratories and maintained on a 12 h light/12 h dark cycle. They were fed Western diet (WD: 42% milkfat, 34% sucrose, and 0.2% cholesterol, Harlan Teklad, TD.88137) or a matched low-fat diet (LFD: 10 kcal % fat, Harlan Teklad TD.08485) that had or did not have dapagliflozin (5 mg/kg per body weight/day) added to them. Following the 26-week treatment period the animals were sacrificed. 

Food intake and body weight were measured every 2 weeks. Mice were placed in metabolic cages for 24-h urine collection for measurement of volume, creatinine, and albumin content. Blood pressure was measured using the tail artery technique with the BP-2000 Blood Pressure Analysis SystemTM (Visitech Systems, Inc., Apex, NC, USA). At the end of the study period, the mice were anesthetized, and blood was drawn for measurement of glucose, triglyceride, and cholesterol.

Animal studies and related protocols were approved by the Animal Care and Use Committee (B-63914(11)2E, 3 September 2014) at the University of Colorado Denver. All animal experimentation was conducted in accordance with the Guide for Care and Use of Laboratory Animals (National Institutes of Health, Bethesda, MD, USA).

### 4.2. Blood and Urine Chemistry

Fasting plasma glucose levels were measured with a Glucometer (Elite XL; Bayer, Tarrytown, NY, USA). Fasting plasma lipid levels were measured with a commercially available kit (Wako Chemical, Richmond, VA, USA). Urine albumin and creatinine concentrations were determined using kits from Exocell (Philadelphia, PA, USA). 

### 4.3. RNA Extraction and Quantitative Real-Time PCR

Total RNA was isolated from the kidneys using the SV total RNA isolation system from Promega (Madison, WI, USA), and cDNA was synthesized using reverse transcript reagents from Bio-Rad Laboratories (Hercules, CA, USA). The mRNA level was quantified using a Bio-Rad iCyCler real-time PCR machine. 18s was used as an internal control, and the amount of RNA was calculated by the comparative threshold cycle (CT) method as recommended by the manufacturer. All the data were calculated from triplicate reactions. Primer sequences are listed in Table S1.

### 4.4. Western Blotting

The protein concentration was measured by the BCA assay (Thermo Fisher Scientic, Waltham, MA, USA). Equal amounts of total protein were separated by SDS-PAGE gels and transferred onto PVDF membranes. Primary antibodies used include Nox2 (ab131083; Abcam, Cambridge, MA, USA; 1:1000 dilution), and β-actin (A5316; Sigma-Aldrich, St. Louis, MO, USA; 1:5000 dilution). After HRP-conjugated secondary antibodies, the immune complexes were detected by chemiluminescence captured on UVP Biospectrum 500 Imaging System (Upland, CA, USA) and the densitometry was performed with ImageJ software.

### 4.5. Two Photon Excitation (TPE) and Second Harmonic Generation (SHG) Microscopy

SHG and TPE microscopy for label-free imaging of collagen and related structures was performed using a Zeiss 780 microscope (Carl Zeiss, Jena, Germany) equipped with a Coherent Chameleon Ultra II laser (Coherent, Santa Clara, CA, USA). For SHG, the laser was tuned to 800 nm and laser power of 7% was used throughout all measurements. A filter cube containing a narrow band 390–410 nm emission filter (hq400/20 m-2p, Chroma Technology, Bellows Falls, VT, USA) was used to detect the SHG signal on a non-descanned detector (NDD). A dichroic mirror at 425 nm allows detection of the TPAF signal at 450–700 nm (hq575/250 m-2p, Chroma Technology, Bellows Falls, VT, USA), also with a NDD detector [44].

### 4.6. Coherent Anti-Stokes Raman Scattering (CARS) Microscopy

We performed CARS microscopy for label-free imaging of lipid deposits as we have previously detailed [45].

### 4.7. Histology Staining and Immunofluorescence Microscopy

Sections (4 m thick) cut from 10% formalin-fixed, paraffin-embedded kidney or liver samples were used for hematoxylin and eosin (H&E) staining, Periodic acid-Schiff (PAS) or periodic acid-Schiff (PAS) staining. Histologic images of H&E stained tissue were captured on an Olympus BX51 microscope equipped with a four-megapixel Macrofire digital camera (Optronics; Goleta, CA, USA). For Picrosirius Red (PSR) staining, slides were first immersed in Bouin’s fixative for 30 min and then the hydrated slides were immersed in Sirius Red solution (Direct Red 80 and saturated picric acid, Sigma) and briefly washed in 0.5% acetic acid (Thermo Fisher Scientific, Waltham, MA, USA). Slides stained by PSR were mounted using Cytoseal XYL, a xylene based mounting media (Richard-Allen Scientific, Kalamazoo, MI, USA). 

Frozen sections were used for Oil Red O staining of neutral lipid (cholesterol esters and triglycerides) deposits and immunostaining. For IF microscopy, the kidney and liver tissue sections were stained with antibodies against fibronectin (F7387, Sigma), type IV collagen (SAB4500375, Sigma), nephrin (PRS2265, Sigma), synaptopodin (SAB3500586, Sigma), and CD68 (MCA1957GA, AbD Serotec, Raleigh, NC, USA) and imaged with a laser scanning confocal microscope (LSM 510, Zeiss, Jena, Germany).

All images were cropped and assembled using Photoshop CS2 (Adobe Systems, Inc.; Mountain View, CA, USA). Detailed methods have been reported previously [46].

### 4.8. Statistical Analysis

Results are presented as the mean ± SEM. Data were analyzed by one-way ANOVA with post hoc Bonferroni-Dunn (unless otherwise indicated) for multiple comparisons. Comparisons between two groups were made by unpaired *t*-test. Statistical significance was accepted at the *p* < 0.05 level.

## Figures and Tables

**Table 1:** Metabolic parameters.

	LF	LF + Dapagliflozin	WD	WD + Dapagliflozin
Body Weight (g)	34.81 ± 0.97	34.21 ± 1.18	54.69 ± 1.16 *	50.09 ± 1.73 ^#^
Kidney weight (g)	0.28 ± 0.01	0.29 ± 0.02	0.47 ± 0.03 *	0.42 ± 0.01
Liver weight (g)	1.25 ± 0.02	1.24 ± 0.01	2.24 ± 0.09 *	1.98 ± 0.06 ^#^
Plasma Glucose (mg/dL)	121.7 ± 1.5	119.8 ± 1.2	206.9 ± 3.1 *	183.8 ± 2.7 ^#^
Plasma Cholesterol (mg/dL)	137.9 ± 1.2	128.8 ± 1.5	343.7 ± 4.2 *	260.1 ± 3.8 ^#^
Plasma Triglycerides (mg/dL)	45.7 ± 3.2	44.2 ± 2.1	59.1 ± 6.4 *	46.2 ± 5.3 ^#^
Plasma insulin (ng/mL)	2.8 ± 0.2	2.5 ± 0.2	5.7 ± 0.7 *	4.2 ± 0.5 ^#^
Systolic blood pressure (mmHg)	121.8 ± 7.9	118.9 ± 1.5	131.2 ± 3.2	128.5 ± 3.6
Diastolic blood pressure (mmHg)	78.5 ± 5.8	83.5 ± 7.3	72.5 ± 5.8	86.5 ± 3.1

* vs. Low fat group, ^#^ vs. Western diet group, *p* < 0.05, values are means, ± SEM (*n* = 6 mouse).

## Supplementary Materials

Supplementary materials can be found at www.mdpi.com/1422-0067/19/1/137/s1.
