# Supplementary material for: The Sodium-Glucose Cotransporter 2 Inhibitor Dapagliflozin Prevents Renal and Liver Disease in Western Diet Induced Obesity Mice
Source: Int J Mol Sci. 2018 Jan 3;19(1):137. doi: 10.3390/ijms19010137 (PMC5796086; doi:10.3390/ijms19010137)

Supplementary

The Sodium-Glucose Cotransporter 2 Inhibitor Dapagliflozin Prevents Renal and Liver Disease in Western Diet Induced Obesity Mice

Dong Wang [1,2], Yuhuan Luo [2], Xiaoxin Wang [2,3], David J. Orlicky [2], Komuraiah Myakala [2,3], Pengyuan Yang [1,4] and Moshe Levi [2,3,*]

[1] Institutes of Biomedical Sciences and Department of Chemistry, Fudan University, Shanghai 200032, China; dong.2.wang@ucdenver.edu (D.W.); pyyang@fudan.edu.cn (P.Y.)

[2] Department of Renal & Hypertension, Department of Pediatrics and Department of Pathology, University of Colorado Denver, Anschutz Medical Campus, 12800 19th Ave., Aurora, CO 80045, USA; yuhuan.luo@ucdenver.edu (Y.L.); xiaoxin.wang@gergeotown.edu (X.W.); david.orlicky@ucdenver.edu (D.J.O.); komuraiah.myakala@georgetown.edu (K.M.);

[3] Department of Biochemistry and Molecular & Cellular Biology, Georgetown University, Washington, DC 200072, USA

[4] Department of Systems Biology for Medicine, Basic Medical College, Fudan University, Shanghai 20032, China

* Correspondence: Moshe.Levi@georgetown.edu

Table S1
Real-time PCR primer sequences

Gene	Forward sequence	Reverse sequence
Srebp1c	GCAGCCACCATCTAGCCTG	CAGCAGTGAGTCTGCCTTGAT
NFkb	ATGGCAGACGATGATCCCTAC	CGGAATCGAAATCCCCTCTGTT
ICAM	GTGATGCTCAGGTATCCATCCA	CACAGTTCTCAAAGCACAGCG
MCP1	TAAAAACCTGGATCGGAACCAAA	GCATTAGCTTCAGATTTACGGGT
TLR2	CACCACTGCCCGTAGATGAAG	AGGGTACAGTCGTCGAACTCT
OPN	AGAGCGGTGAGTCTAAGGAGT	TGCCCTTTCCGTTGTTGTCC
TGFb1	CTCCCGTGGCTTCTAGTGC	GCCTTAGTTTGGACAGGATCTG
Nox-2	AGTGCGTGTTGCTCGACAA	GCGGTGTGCAGTGCTATCAT
chrebp	AGATGGAGAACCGACGTATCA	ACTGAGCGTGCTGACAAGTC
TNFa	CAGGCGGTGCCTATGTCTC	CGATCACCCCGAAGTTCAGTAG
IL1B	GCAACTGTTCCTGAACTCAACT	ATCTTTTGGGGTCCGTCAACT
TLR4	GCCTTTCAGGGAATTAAGCTCC	GATCAACCGATGGACGTGTAAA
COL1A1	GCTCCTCTTAGGGGCCACT	CCACGTCTCACCATTGGGG
COL3A1	CTGTAACATGGAAACTGGGGAAA	CCATAGCTGAACTGAAAACCACC
p22phox	TCACCAGGAATTACTACGTCCG	GCTGCCAGCAGATAGATCACA
p40phox	TGTGGTTGGGGCTGAATGTC	CTGAGAAAGGAGAGCAGATTTCG

Figure S1. Picro sirius red staining of kidney sections

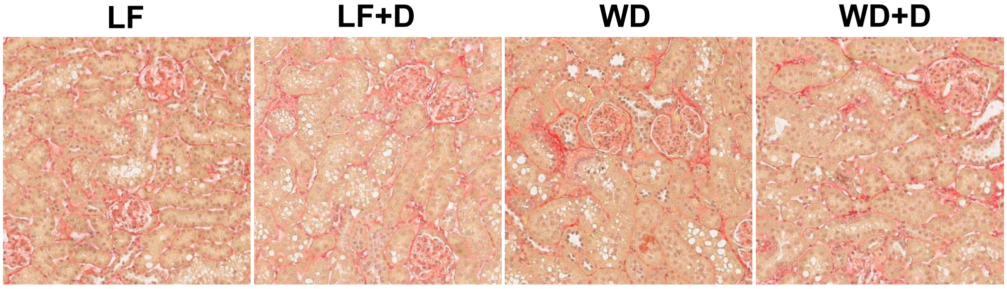

Supplement: Supplementary file 1 [file ijms-19-00137-s001.pdf]
